# Retinal vessel volume reference database derived from volume-rendered optical coherence tomography angiography

**DOI:** 10.1038/s41598-024-53000-8

**Published:** 2024-02-01

**Authors:** Silvia Feu-Basilio, Peter M. Maloca, Pascal Hasler, Hendrik P. N. Scholl, Sara Marin-Martinez, Josep Rosinés-Fonoll, Xavier Suarez-Valero, Michael Reich, Clemens Lange, Catherine Egan, Sandrine Zweifel, Adnan Tufail, Richard F. Spaide, Javier Zarranz-Ventura

**Affiliations:** 1 Hospital Clínic de Barcelona, University of Barcelona, Carrer de Sabino Arana, 1, 08028 Barcelona, Spain; 2 Institute of Molecular and Clinical Ophthalmology Basel, 4031 Basel, Switzerland; 3 Department of Ophthalmology, University Hospital Basel, 4031 Basel, Switzerland; 4 Moorfields Eye Hospital NHS Foundation Trust, London, EC1V 2PD UK; 5 Faculty of Medicine, Eye Center, Albert-Ludwig University Freiburg, 79085 Freiburg, Germany; 6 Augenärzte Am Städel, Hans-Thoma-Strasse 24, 60596 Frankfurt Am Main, Germany; 7 Department of Ophthalmology, St. Franziskus Hospital, 48145 Münster, Germany; 8 Department of Ophthalmology, University Hospital Zurich, 8006 Zurich, Switzerland; 9 University of Zurich, 8006 Zurich, Switzerland; 10 Vitreous, Retina, Macula Consultants of New York, New York, NY USA; 11 Institut de Investigacions Biomediques August Pi i Sunyer (IDIBAPS), 08036 Barcelona, Spain

**Keywords:** Eye diseases, Imaging techniques, Biomarkers, Medical research

## Abstract

Optical coherence tomography angiography (OCTA) enables three-dimensional reconstruction of the functional blood vessels in the retina. Therefore, it enables the quantification of 3D retinal vessel parameters such as surface area and vessel volume. In spite of the widespread use of OCTA, no representative volume-rendered vessel volume (VV) data are published to date. In this study, OCTA 3 × 3 mm macular cubes were processed with volume-rendering techniques to measure VV in 203 eyes from 107 healthy volunteers. Generalized linear models (GLM) were constructed to assess the impact of age, gender, visual acuity (VA), spherical equivalent (SE), and axial length (AL) on VV. Overall mean VV was 0.23 ± 0.05mm^3^. Age and axial length showed a negative correlation with VV. However, GLM model analysis found that AL exerted the most pronounced influence on VV. No statistically significant associations were identified between gender or between left and right eyes. This is the first study to assess 3D OCTA VV and its naturally occurring variations in a large series of healthy subjects. It offers novel insights into the characterization of normal retinal vascular anatomy in healthy individuals, contributing to a valuable reference for future research in this field.

## Introduction

Optical coherence tomography angiography (OCTA) is an imaging technique that uses the variation in signal intensity caused by moving blood particles, such as erythrocytes, as the contrast mechanism for the visualization of functional blood vessels in the eye^1,2^. Therefore, OCTA enables the reconstruction of retinal microvascular flow maps, as a beneficial imaging tool in a series of microvascular pathologies, such as diabetic retinopathy^3,4^. In diabetic retinopathy, OCTA has helped to document disease progression and to assess risk stratification by providing two-dimensional capillary perfusion density maps for the quantification of the superficial and deep capillary layers. The quantification of the rich granular data provided by OCTA images has made it possible to expand a further field of research allegedly called OCTA angyolitics^5^. In addition, OCTA metrics of the retinal vasculature allows investigating the correlation of these findings with microvascular damage in other organs such as the kidney or the previous glycemic control^6–8^.

However, although OCTA by itself is a three-dimensional imaging technique, for the purpose of comprehensibility, most commercial instruments offer a limited two-dimensional-en face-representation of OCTA data. The latter is the display method most currently provided for both clinical practice and clinical research^2,9–11^. En face imaging might be more handy, but it actually only depicts a small portion of the real-world volume data of retinal vessels. This can result in the underestimation of tissue perfusion, lack of visualization of true flow or misinterpretation due to segmentation errors for specific layers within the retina^12^.

Volume rendering of the underlying three-dimensional OCTA data is an exciting avenue of research, avoiding many of these problems as it allows visualization of all layers of flow in the retina, comprehending the spatial interrelationship between certain lesions^12^. In other words, volume-rendered OCTA enables to further adequately model the physical characteristics of the retinal vessel architecture which is not made possible with previous retinal imaging modalities such as invasive fundus fluorescein angiography or indocyanine green angiography. The detectable 3D OCTA volume of retina blood vessels refers largely to the total space occupied by the blood within the vessel^13,14^. This volume can vary depending on the type of vessel (artery, vein, capillary) and its size^13,14^. Arteries, which carry blood away from the heart, typically have a smaller volume compared to veins, which return blood to the heart. This is because arteries have thicker walls and a smaller lumen (inner space) relative to their outer diameter. Capillaries, on the other hand, are the smallest and most numerous blood vessels. They have a tiny volume individually, but their collective volume is substantial due to their sheer number. There is an inverse correlation between the surface area^9^ and the volume of blood vessels. As blood vessels branch into smaller vessels (arteries to arterioles to capillaries), their total surface area increases dramatically, while their total volume decreases. In this context, it is worth to note that the relationship between the vessel surface area and volume of blood vessels is an important consideration in biology and physiology, as it has significant implications for the exchange of gases, nutrients, and waste products within the circulatory system^2^.

Therefore, the aim of this study was to investigate the natural variation of VV in a relatively large healthy cohort and to determine its association with the demographic features and a series of clinically relevant characteristics (i.e. sex, age, spherical equivalent -SE-, axial length -AL- and visual acuity -VA-).

## Results

### Summary statistics and data plots

The OCTA measurements from 203 eyes (102 left and 101 right eyes) of 107 subjects (61.7% female) could be included, being 97.5% of the eyes phakic. Twenty-three eyes were excluded because of the presence of coronary heart disease (n = 2), peripheral vascular disease (n = 10), high myopia (n = 2), amblyopia (n = 6), or cataract (n = 3).

**Figure 1:**
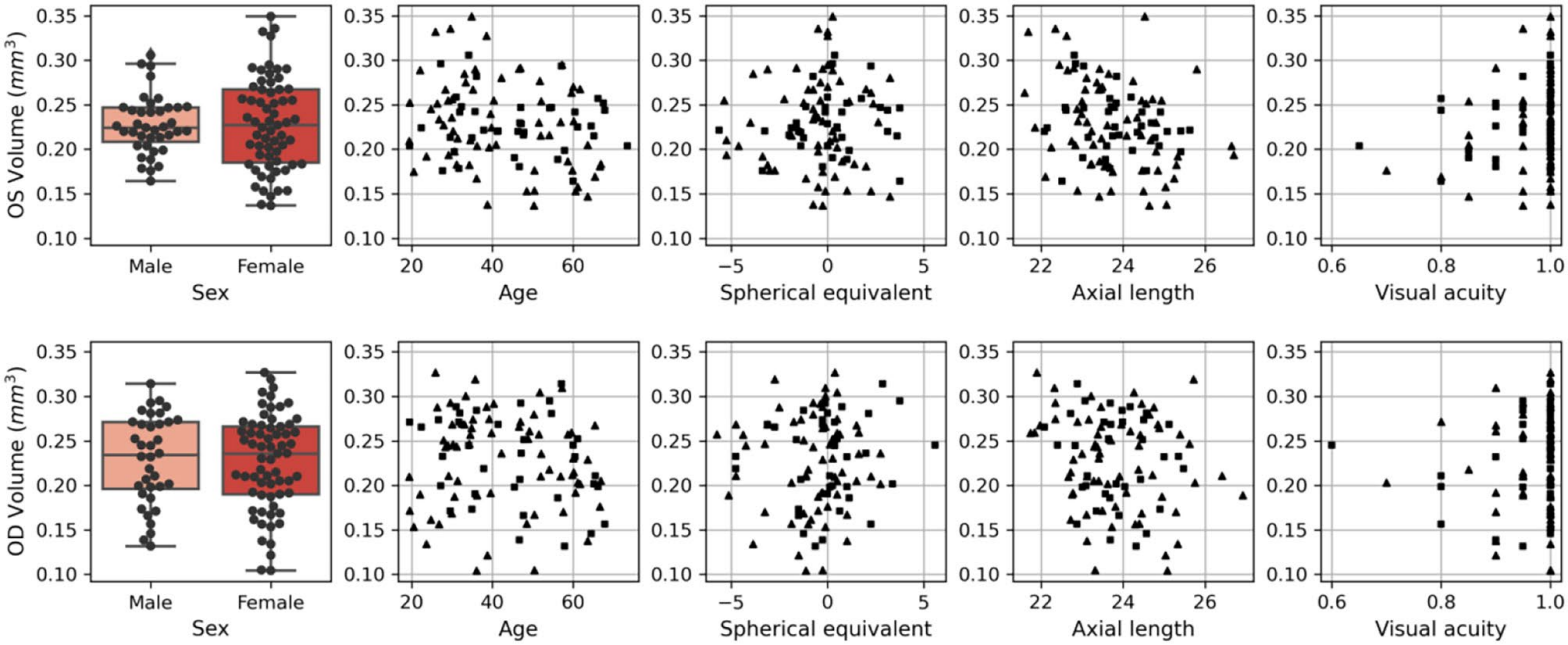
Univariate box plots and scatter plots of 3D OCTA vessel volume metrics. Plots in the first row are based on data from the left eyes (OS). Plots in the second row are based on data from the right eyes (OD). Boxplots are grouped by sex. Scatter plots are drawn for age, spherical equivalent, axial length, and visual acuity. Males (females) are indicated by squares (triangles) in the scatter plots. Optical coherence tomography angiography retinal vessel volume measurements are in mm^3^.

The summary statistics are presented in Table 1 and the data are plotted in Fig. 1. Overall mean VV was 0.23 mm^3^ (SD 0.05). The distribution of VV values ranged from 0.10 to 0.35 mm^3^. To avoid correlation effects between the eyes, further results are separated by right and left eyes. All the measured raw data are summarized in Supplementary raw data (S1) (right eyes) and Supplementary raw data (S2) (left eyes).

**Table 1:** Summary statistics of total retinal vessel volume and parameters.

	Age	SE	AL	VA	VV overall	VV male left	VV female left	VV male right	VV female right
Count	203	197	200	202	203	39	63	36	65
Mean	43.39	− 0.41	23.8	0.97	0.23	0.23	0.23	0.23	0.23
Std	14.05	2.04	1.03	0.07	0.05	0.03	0.05	0.05	0.05
Min	19.37	− 5.75	21.59	0.60	0.10	0.16	0.14	0.13	0.10
25%	31.08	− 1.38	23.07	0.95	0.19	0.21	0.19	0.20	0.19
50%	41.25	− 0.25	23.68	1.00	0.23	0.22	0.23	0.23	0.24
75%	56.84	0.5	24.49	1.00	0.27	0.25	0.27	0.27	0.27
Max	73.49	5.59	26.92	1.00	0.35	0.31	0.35	0.31	0.33

Summary statistics are calculated from 203 eyes (102 left and 101 right). Count indicates the number of eyes for which the respective measurements were available. Mean, std, min, 25%, 50%, 75% and max indicate mean, standard deviation, minimum, 1st quartile, 2nd quartile, 3rd quartile, and maximum, respectively. Age is measured in years. SE, spherical equivalent (diopters); AL, axial length (mm); VA, visual acuity (decimal scale); VV, OCTA vessel volume (mm^3^).

### Correlation analysis

Pearson correlation coefficients between VV and age were  − 0.11 and  − 0.20 for right and left eyes, respectively. Testing for non-correlation revealed no significant correlation in right eyes (*p* = 0.257) but a significant negative correlation in left eyes (*p* = 0.041).

Pearson correlation coefficients between VV and AL were  − 0.19 and  − 0.29 with *p*-values 0.063 and 0.004 in right and left eyes, respectively.

### Generalized linear model (GLM) analysis

GLM models were constructed to evaluate the effects of ocular parameters (age, sex, AL and VA) on VV. Results (Table 2) showed that the main effect of AL had a significant effect on VV on both left (*p* < 0.01) and right eyes (*p* < 0.05). For the left eyes only, the main effect of VA had a significant effect (*p* < 0.05) on VV. On the other hand, a two-way interaction, sex: SE, was found to have a significant effect (*p* < 0.05) on right eyes only. No other effects and two-way interactions were significant in either eye at a significance level of 0.05.

**Table 2:** Generalized linear model (GLM) analysis results.

Effect	Right eyes			Left eyes		
	F value	Pr (> F)	Sign	F value	Pr (> F)	Sign
Age	0.4551	0.50186		2.1512	0.1463308	
Sex	0.2747	0.60161		0.9389	0.3354543	
SE	0.0805	0.77736		1.2825	0.2607822	
AL	4.0088	0.04861	*	13.6097	0.0004061	***
VA	0.6588	0.41935		4.355	0.0400442	*
Age:Sex	3.0123	0.08644		2.0743	0.1536501	
Age:SE	0.4186	0.51946		0.3454	0.5583766	
Age:AL	0.3268	0.56916		0.292	0.5904485	
Age:VA	0.0697	0.79244		0.2403	0.6253133	
Sex:SE	4.4824	0.03731	*	0.7343	0.3940192	
Sex:AL	2.0398	0.15708		0.9675	0.328236	
Sex:VA	0.025	0.8747		0.0207	0.8859757	
SE:AL	0.5212	0.47242		0.03	0.8629057	
SE:VA	0.2447	0.62218		0.0358	0.8503498	
AL:VA	0.4269	0.51538		0.1837	0.6693748	

A GLM analysis was performed for the left and right eyes separately. The main effects of the independent variables, age, sex, spherical equivalent (SE), axial length (AL), and visual acuity (VA), and all the possible two-way interactions are showed. *p*-values were calculated using the F-test statistic. The “Sign” columns indicate significance codes: 0, ‘***’; 0.001, ‘**’; 0.01, ‘*’; 0.05; and 0.1, 1.

### Right vs left eyes

A paired two-sided *t*-test on VV was performed to assess whether there was a significant difference in left vs right eyes. The result was *p*-value of 0.70 (value of *t*-test statistic, 0.38). Therefore, it was concluded that there was no significant difference in VV between the left and right eyes.

### Correlation analysis between surface and volume

Our group previously studied 3D OCTA total surface area (i.e. vessel surface) using the same automatic technology for 3D rendering of OCTA and the same population sample as in this study^9^. We performed a correlation analysis between Surface and VV. Pearson correlation coefficients between vessel surface and VV were 0.98 in left eyes and 0.99 in right eyes.

## Discussion

This study reports a normative database of VV in healthy controls and describes that this novel biomarker shows existing associations with age and ocular parameters such as axial length in healthy subjects. Interestingly, we observed that even within this healthy and relatively large cohort, natural variations in VV of over 30 per cent were already detected. These results reinforce the notion that when recruiting for OCTA studies, attention must be paid to the composition of the participants to avoid bias in the results due to natural fluctuations.

OCTA is an imaging technique that is revolutionizing the diagnosis and monitoring of multiple ophthalmologic diseases. The reason why retinal vessel volumes are important in the current study can be challenged. Previous methods such as FA or ICG have demonstrated their usefulness for clinical practice but never reveal the entire vessel architecture. Also, they only provide qualitative values of the vasculature so that a comparative assessment is difficult due to the lack of quantifiability. In the former two-dimensional OCTA method, the quantification of the vessels is based only on measurements of their 2D images, causing that only a small part of a vessel can be measured effectively since vessels are three-dimensional elements. In contrast, the current study allows the measurement of the OCTA derived endoluminal blood flow in three-dimensions, which makes possible in a previously unimagined approximation to effectively assess naturally existing vessel structures. Thus, both elements, OCTA VV and OCTA vessel surface may be useful parameters in the future, as it is possible that certain disease processes may attack them differently^2,15^. For example, in diabetic retinopathy, it is conceivable that as a result of the loss of pericytes, the vessel surface may be affected differently than the vessel volume.

These results may offer further insights in the microvascular status of the retinal vessels, beyond the standard 2D metrics commonly used to date in clinical care and research as reported in the literature. OCTA demonstrated promise in various retinal diseases such as diabetic retinopathy, age-related macular degeneration, and retinal vascular occlusions, and also was proven useful in anterior segment and optic nerve related alterations^1,3,4,6,10–12,16–18^. However, most of these studies were carried out using the standard two-dimensional en face representation. As previously described, the concept of providing only 2D OCTA data reflects only a reduced capability of data representation, as retinal vessels are by themselves three-dimensional structures^2,9^. The relevance of volume rendering techniques lie in the possibility to bring along novel three-dimensional parameters to study the status of the retinal microvasculature, such as vessel surface or VV, which can be altered differentially in multiple diseases such as diabetic retinopathy or Stargardt disease^2,15^. The goal of this study was to highlight the role of 3D OCTA as an objective tool to broaden our understanding of the retina by using volume rendering of OCTA cubes to measure VV in a large series of healthy subjects and assessing its naturally occurring variations.

To our knowledge this is the first study to measure 3D OCTA VV in such a large series of healthy subjects. Indeed, this parameter has only been previously reported in short series of healthy subjects^2,19,20^. Maloca et al.^2^ studied 26 eyes of 13 healthy individuals (all female) and reported an overall average VV of 0.49 ± 0.09 mm^3^. Borrelli et al.^19^ analyzed 15 eyes of 15 healthy subjects (all female) and found a mean VV of 0.32 ± 0.05 mm^3^, and in a different study examined 35 eyes of healthy controls (19 females) and presented a mean VV of 0.29 ± 0.04 mm^3^^20^. Such different results in mean VV may be due to the small sample size used in these studies and its lesser representativeness with the general population (in the first two cited papers healthy subjects were all female). All compared their results with short series of diabetic patients and found that VV was reduced in diabetic patients. However, correlations of VV with ocular parameters of interest such as age, sex, VA, SE or AL were not explored in these studies.

We explored potential associations of 3D OCTA VV with demographics and clinical parameters recorded in our series. We observed a significant negative correlation between VV and age and AL, consistently demonstrated regardless of eye laterality for right and left eyes. This association remained significant for AL after controlling for potential confounders in the GLM analysis. This finding suggests that VV is directly correlated with the AL of the studied eyes and highlights the relevance of reporting this specific data simultaneously to VV, as this may reflect possible magnification effects that may influence the 3D OCTA metrics provided.

These findings are in accordance with the correlation and GLM analyses conducted in our previous paper investigating vessel surface in 3D OCTA cube scans using the same automatic technology for 3D rendering of OCTA and population sample as in the present study^9^. In this report, we evaluated the effect of AL, VA, SE, sex and age on 3D OCTA total vessel surface area. Similar to the main findings reported here, AL showed a significant association with the total vessel surface observed in healthy eyes. Due to the strong association and high magnitude of the correlation observed in both cases, VV and vessel surface seem to behave in a similar fashion and should be considered reliable 3D OCTA parameters to describe the microvascular status of healthy patients.

This study has a number of limitations. First, our study cohort includes a limited series of relatively young patients, which may have tampered our ability to identify potential physiological changes in VV due to aging. Our series also presented a slight female preponderance, which may have also affected the inter-gender comparison although the data do not suggest any impact of sex. Second, OCTA data cubes were exported as raw files so projection artifact removal^21^ was not provided by the manufacturer’s software. Third, a possible ocular magnification effect may have influenced the measurements provided. There are no published international standards to control for this factor, and some approximations have been conducted using the axial length as covariable^22,23^. To investigate further this matter, we conducted a specific analysis with a correction based on Littmann’s method and Bennett’s formula, detailed in the Supplementary raw data S1 and S2. And fourth, no reproducibility test was specifically conducted for the current VV measurements performed in this study. However, the reproducibility of the volume rendering OCTA method employed has been previously validated^2^ and is comparable to standard enface OCTA methods^24,25^.

In summary, blood vessels are structured to maximize surface area for efficient exchange while minimizing volume to maintain pressure and flow. This arrangement is essential for the proper functioning of the circulatory system and the delivery of oxygen and nutrients to cells throughout the body. This study shows for the first time in a large number of healthy subjects that 3D OCTA can approximate the effective retinal blood column and that a relatively large natural variability has to be taken into account. We provide objective data about the normal variation of VV metrics observed in a large series of healthy subjects, and investigate its correlation with clinical parameters of interest such as age, sex, VA, SE and AL. The clinical value of this parameter will be investigated in future studies directed to evaluate its relevance and potential implications in the pathophysiology of different retinal diseases, such as diabetic retinopathy.

## Methods

### Study design and study protocol

Cross sectional, exploratory study of a control cohort recruited prospectively in a 24-month period with collection of OCTA images and relevant ocular and systemic clinical data as part of a larger prospective OCTA trial (ClinicalTrials.gov, trial number NCT03422965)^16^. This project was approved by the Institutional Review Board of Hospital Clinic of Barcelona (HCB/2016/0216). Written informed consent was obtained for all participants. All research was performed in accordance with the Declaration of Helsinki.

### Inclusion and exclusion criteria

Controls were collected from healthy volunteers recruited in the general population after social media campaigns supported by the Communications department of the hospital. Inclusion criteria were age over 18 years in both sexes, ability to follow instructions and to consciously sign informed consent. Exclusion criteria include concomitant ocular pathology, macular edema, presence of macular cysts, previous ocular surgery, previous macular laser, previous ocular treatment including intravitreal therapy, media opacities and inability to perform complete ocular examinations including retinal imaging (OCT, OCTA, fundus retinographies, biometry, etc.), as well as inability to give written informed consent to participate in the study.

### Data collection

All participants underwent a complete ocular examination, as described elsewhere^16^. Relevant ocular clinical data collected included best-corrected visual acuity (BCVA), spherical equivalent, slit-lamp biomicroscopy, intraocular pressure measurement, retinal fundus exam and axial length (IOL Master 500, Carl Zeiss Meditec, Dublin, CA). Systemic clinical data collected included age, sex, smoking habit, systolic and diastolic blood pressure, height, weight, body mass index (BMI). A comprehensive battery of OCT and OCTA images was captured using the Cirrus OCT device (Carl Zeiss Meditec, Dublin, CA) and included an OCTA 3 × 3 × 2 mm macular scan. OCTA and structural OCT quantitative parameters were determined using the built-in commercial software (Angioplex Metrics) and included vessel density (VD), perfusion density (PD), foveal avascular zone (FAZ) area, perimeter and circularity, central retinal thickness (CRT), macular volume (MV) and average retinal thickness (ART).

### Image analysis and 3D volume rendering

**Figure 2:**
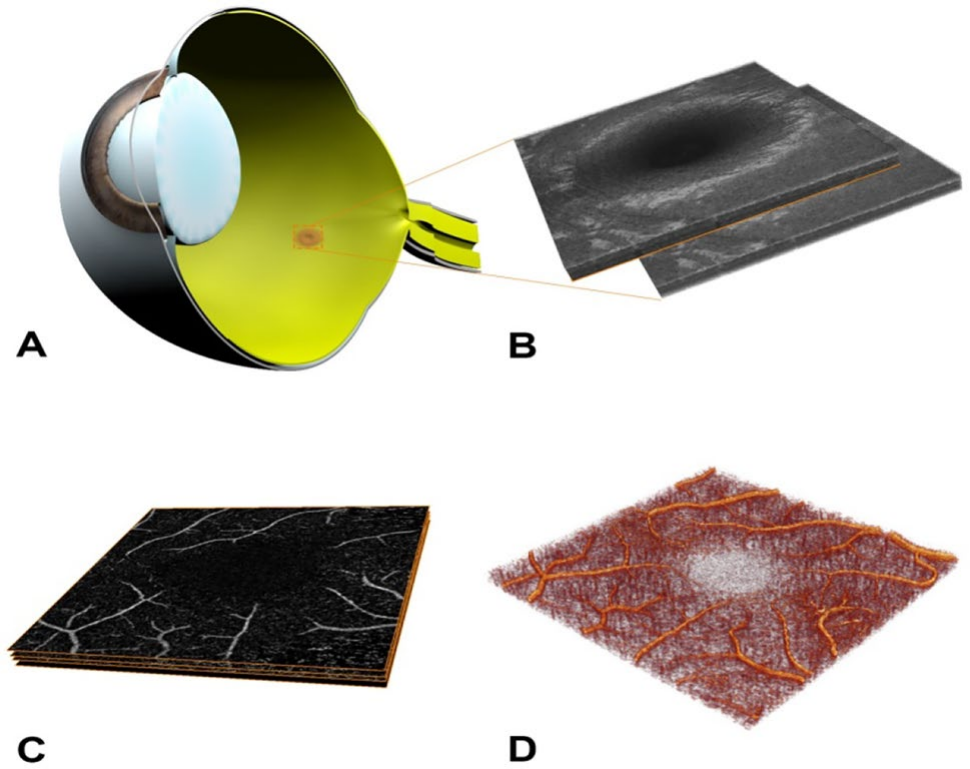
Image processing of 3D volume-rendered OCTA method. Quantification of vessel volume from 3D-volume rendering OCTA cube reconstructions. (**A**) The OCT scan beam was centered over the fovea. (**B**) Multiple volumes were obtained from the OCT scan and the OCTA cross-sectional images were created using the manufacturer’s software (**C**). From these, a volume rendered OCTA was produced (**D**) so that the OCTA vessel volume could be calculated per volume. This figure has been produced with Cinema 4D Release 20 (Build RB255810, licence 14004015757-XRPV, Maxon Computer GmbH, Bad Homburg, Germany, and finalised with Adobe Photoshop (Creative Cloud, Version 25.3.1, Licence ID C5004899101EDCH, Adobe Systems Incorporated, San Jose, US).

Original raw 3 × 3 mm OCTA cubes data were exported from the OCT device as proprietary data in .img image format using the built-in manufacturer's software, which did not allow for direct projection artifact removal. Using a custom written script, images were automatically converted as en face .bmp sequence. Image post-processing included an automatic script written in Matlab R2017a (MathWorks Inc., Natick, USA) to separate the retinal flow signal and make it suitable for measurement of the total three-dimensional vessel volume (Fig. 2). Details of this method, including its reproducibility, have been previously published by our group^2^.

### Statistical analysis

Qualitative variables were described using absolute frequencies and percentages. The description of quantitative variables was performed using the mean, standard deviation (SD), minimum, 1st quartile, 2nd quartile, 3rd quartile, and maximum. Summary statistics were calculated in Python v3.8^26^ with pandas v1.1^27^ and boxplots were generated in Python with Matplotlib v3.3^28^. To account for possible ocular magnification effect, a specific analysis with a correction based on Littmann’s method and Bennett’s formula^22,23^ was also performed, detailed in Supplementary raw data S1 and S2. Pearson correlation coefficients were investigated for right and left eyes separately and calculated in Python v3.8 with scipy v1.6^29^. Generalized linear models were created to evaluate potential existing relationships between VV and ocular parameters of interest. The GLM analysis was performed, on the left and right eyes separately, in R v3.6^30^ with car v3.0^31^ using the type II sum of squares estimation method. For all the tests, *p*-values < 0.05 were considered statistically significant.

### Supplementary Information

Supplementary Information 1.

Supplementary Information 2.

## Data Availability

The datasets generated and analyzed during the current study are available in Supplementary raw data S1 for the right eyes and Supplementary raw data S2 for the left eyes.
